# Supplementary material for: SARS-CoV-2 N protein recruits G3BP to double membrane vesicles to promote translation of viral mRNAs
Source: Nat Commun. 2024 Dec 5;15:10607. doi: 10.1038/s41467-024-54996-3 (PMC11621422; doi:10.1038/s41467-024-54996-3)
Supplement: Supplementary file 1 — Supplementary Information [file 41467_2024_54996_MOESM1_ESM.pdf]

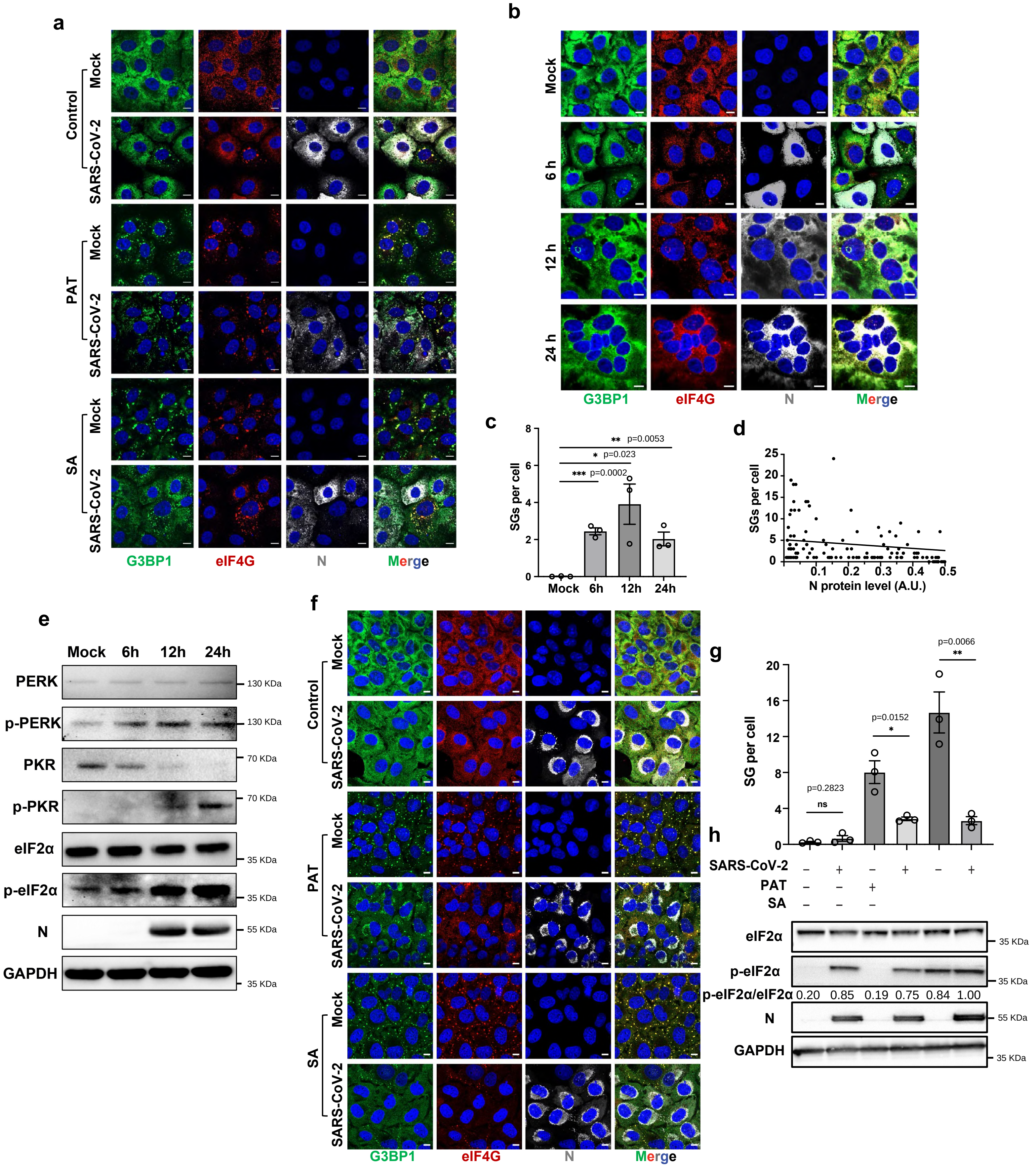

Supplementary Fig. 1. SARS-CoV-2 infection inhibited SG formation independently of eIF2α. (a) VeroE6 cells were infected with SARS-CoV-2 at 0.5 MOI. At 6 hpi, cells were stressed with SA or PAT for 1h before fixation. Cells were stained for indicated antibodies and representative images from three independent experiments are shown. Scale bar = 10 µm. (b) U2OS-ACE2 cells were mock infected or infected with SARS-CoV-2 WT at 0.5 MOI. Cells were fixed at 6, 12, 24 h and stained using indicated antibodies. Scale bar = 10 µm. (c-d) Quantification of SG foci and N protein intensity from (a) were performed by CellProfiler. (c) Bars represent mean +/– SEM for n=3 biological replicates with each dot representing the mean of 30 cells. (d) n=298 cells. The correlation of N protein level and SG numbers per cell was calculated by Pearson correlation coefficient r = - 0.3, $p < 0.0001$ (two-tailed). (e) U2OS-ACE2 cells were infected with SARS-CoV-2 at 0.5 MOI for 6, 12, 24 h, respectively. Cell lysates were separated by SDS-PAGE and probed with indicated antibodies. Representative images from three independent experiments are shown. (f) U2OS-ACE2 cells were mock infected or infected with SARS-CoV-2 at 0.5 MOI. At 6 hpi, cells were stressed with SA or PAT for 1h before fixation and staining with indicated antibodies. Representative images from three independent experiments are shown. Scale bar = 10 µm. (g) Quantification of SG foci in (f) was performed by CellProfiler. Bars represent mean +/– SEM for n=3 biological replicates with each dot representing the mean of 25 cells. (h) Cells were lysed for immunoblotting with indicated antibodies, and representative images from three independent experiments are shown.

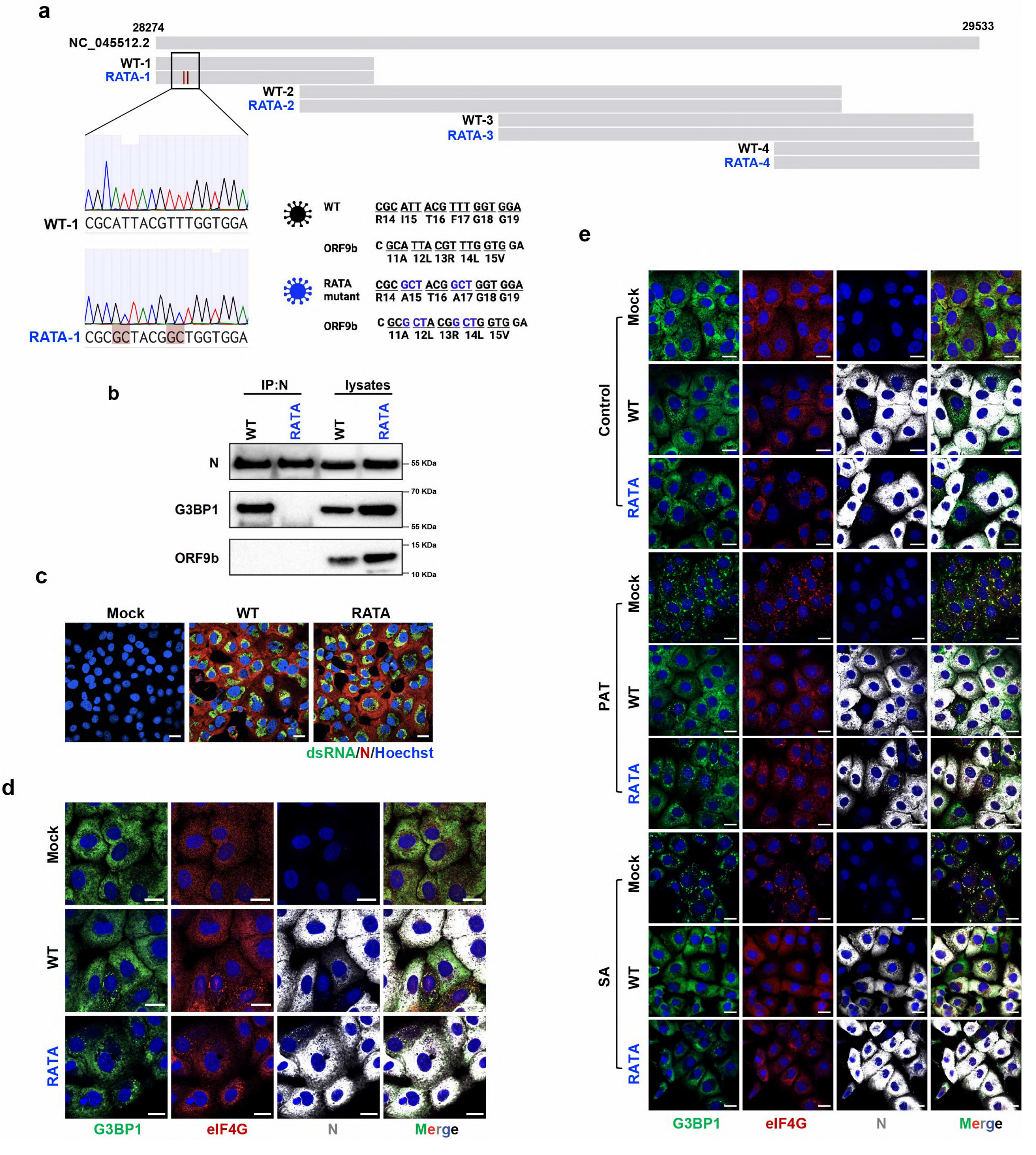

Supplementary Fig. 2. SARS-CoV-2 WT and RATA mutant. (a) Sanger sequencing of RATA P0 stock confirmed that no additional mutations were present in the N/ORF9b gene, created in BioRender. The alignment of sequenced data was perform ed using Benchling. The template sequence NC_045512.2 (28274-29533) served as the reference sequence. (b) VeroE6 were i nfected with SARS-CoV-2 or RATA mutant (P0 stock) for 48 h, cells were lysed and immunocipitated using N antibody for immu noblotting with indicated antibodies. Representative images from three independent experiments are shown. (c) VeroE6 cells were i nfected with P0 stock of rescued WT or RATA mutant for 24 h, then fixed and stained with dsRNA (green) and N(red), Hoechst (blue). Representative images from three independent experiments are shown. Scale bar = 20 µm. (d) VeroE6 cells were infected with WT virus or RATA mutant at 0.5 MOI for 6 h and then fixed and stained with indicated antibodies. Represen tative images from three independent experiments are shown. Scale bar = 20 µm. (e) VeroE6 cells were infected with SARS-C oV-2 WT or RATA at 0.5 MOI. At 6 hpi, cells were stressed with SA or PAT for 1h before fixation and staining with indicated antibodies. Representative images from three independent experiments are shown. Scale bar = 20 µm.

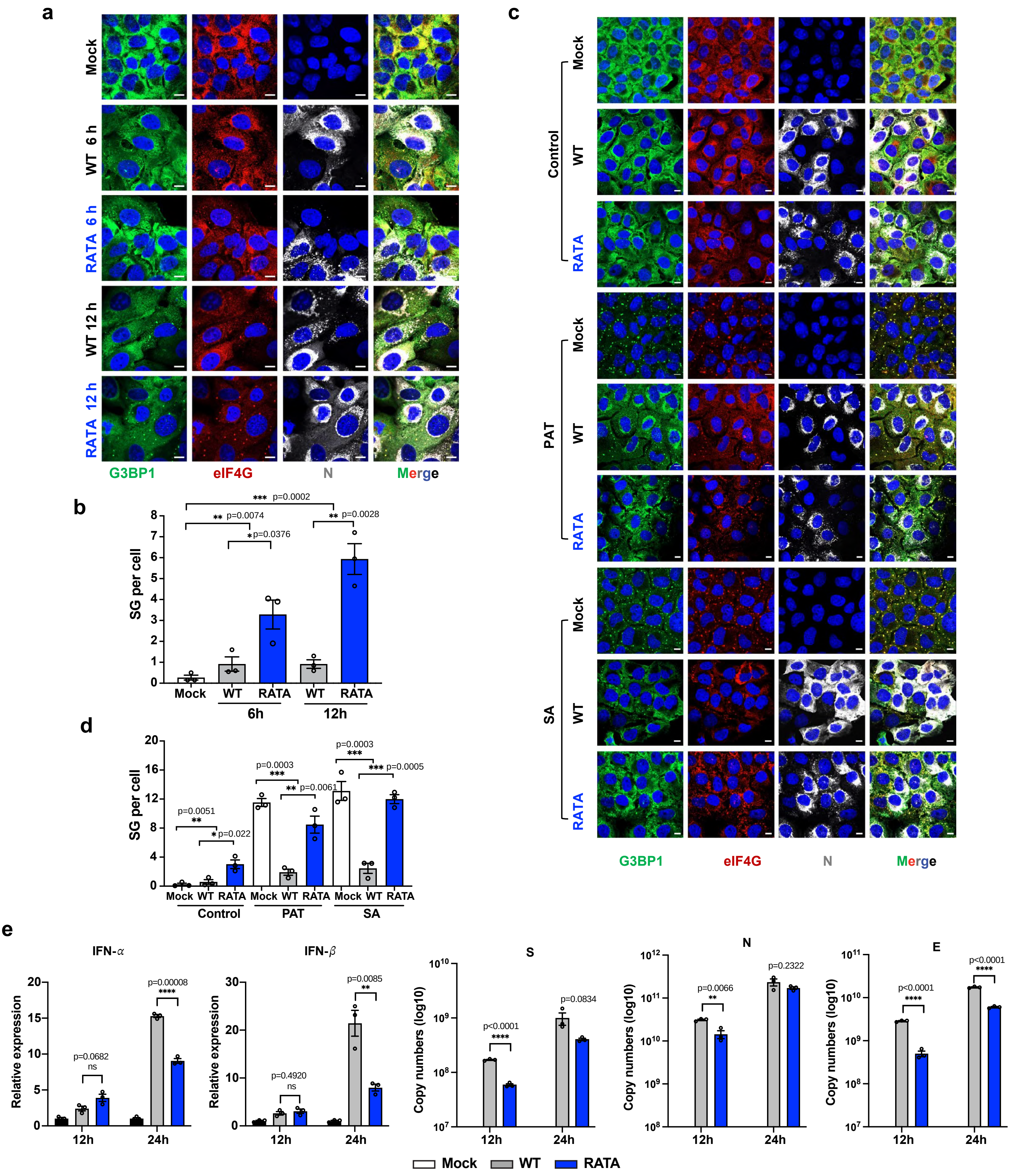

Supplementary Fig. 3. SARS-CoV-2 RATA is defective in SG inhibition and is attenuated in multiple cell lines. (a) U2OS-ACE2 cells were infected with SARS-CoV-2 WT or RATA at 0.5 MOI for 6 or 12 h, and then fixed and stained with indicated antibodies. Representative images from three independent experiments are shown. Scale bar = 10 µm. (b) Quantification of SG foci from (a) was performed using CellProfiler. Bars represent mean +/– SEM for n=3 biological replicates with each dot representing the mean of 20 cells. (c) U2OS-ACE2 cells were infected with WT virus or RATA mutant at 0.5 MOI. At 6 hpi, cells were stressed with SA or PAT for 1h before fixation and staining with indicated antibodies. Representative images from three independent experiments are shown. Scale bar = 10 µm. (d) Quantification of SG foci in (c) was performed using CellProfiler. Bars represent mean +/– SEM for n=3 biological replicates with each dot representing the mean of 30 cells. (e) RT-qPCR analysis for quantification of IFN-α, IFN-β expression and viral genome copies (S, N, E) from U2OS-ACE2 cells infected with SARS-CoV-2 WT or SARS-CoV-2 RATA at 0.05 MOI (n=3 biological replicates).

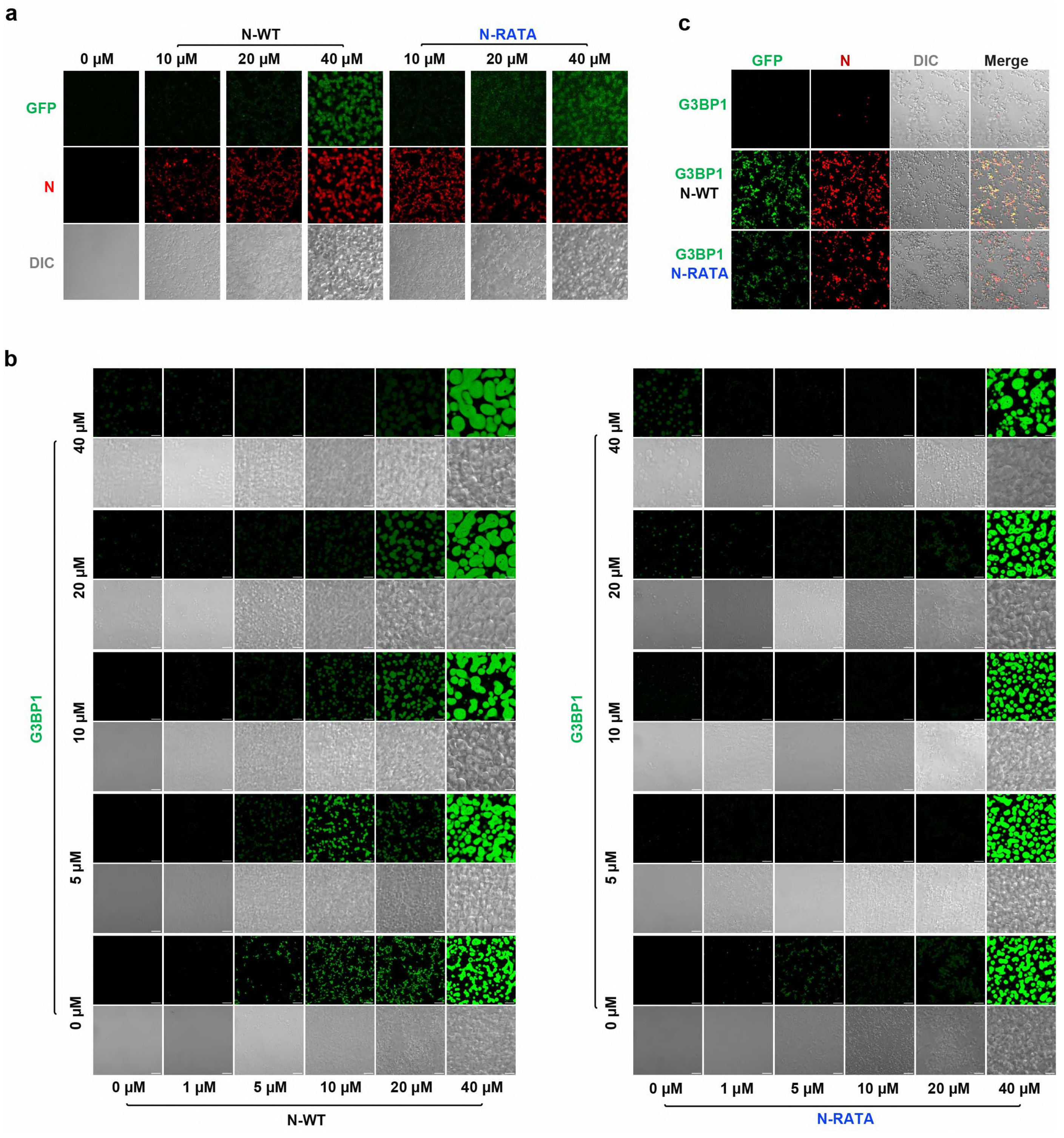

Supplementary Fig. 4. G3BP1 facilitates LLPS of N, and G3BP-N induces distinct lysate granules. (a) Purified N-WT or N-RATA protein was added at varying concentrations to lysates from ΔΔGFP cells (lacking both G3BP1 and G3BP2, and stably expressing GFP alone). N specific antibodies conjugated to Alexa Fluor 647 was used to visualize N protein. Representative images from three independent experiments are shown. (b) Phase separation behaviours of the N-WT or N-RATA with increasing concentrations of purified G3BP1 in ΔΔGFP-G1-WT cell lysate. (c) Addition of purified G3BP1 (20 μM), N-WT (10 μM) or N-RATA (10 μM), RNase A into ΔΔGFP-G1-WT cell lysate. Fluor-conjugated antibodies were used to visualize N (red), eIF4G (yellow), and GFP (green) indicated GFP-G3BP1. Representative images from three independent experiments are shown. Scale bar = 10 μm.

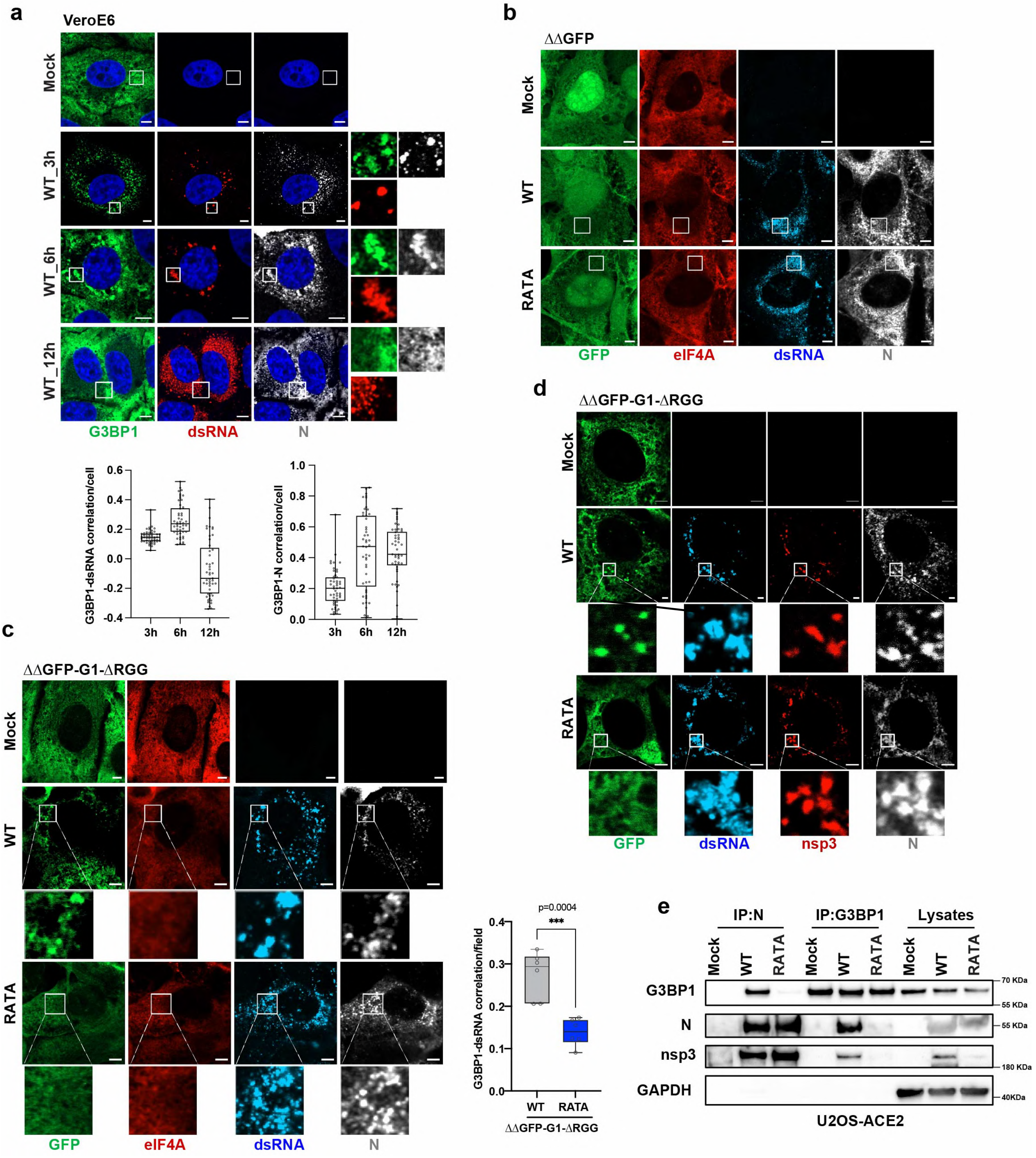

Supplementary Fig. 5. N recruits G3BP1 to RTC early in infection via interaction with pore protein nsp3. (a) VeroE6 cells were infected with WT SARS-CoV-2 at 0.5 MOI. Cells were fixed at indicated time point and stained for G3BP1 (green), dsRNA (red) and N (grey), Hoechst (blue). Representative images from three independent experiments are shown. Scale bar = 5 µm. Correlations of G3BP1-dsRNA, or G3BP1-N were calculated in CellProfiler based on Pearson's correlation coefficient for n = 49 infected cells. (b-d) Indicated cell lines were infected with SARS-CoV-2 WT or RATA at 0.5 MOI for 6 h. Cells were fixed and stained for dsRNA (blue), N (grey) and eIF4A (b-c) or nsp3 (d). Representative images from three independent experiments are shown. Scale bar = 5 µm. Pearson's correlation coefficients for colocalization of G3BP1 and dsRNA in ΔΔGFP-G1-ΔRGG cells were analyzed in CellProfiler (n = 6 dsRNA-positive fields). (e) U2OS-ACE2 cells were infected with WT virus or RATA mutant at 0.01 MOI for 24 h. Cells were lysed and immunoprecipitated with G3BP1 or N antibody for immunoblotting as indicated. Representative images from three independent experiments are shown.

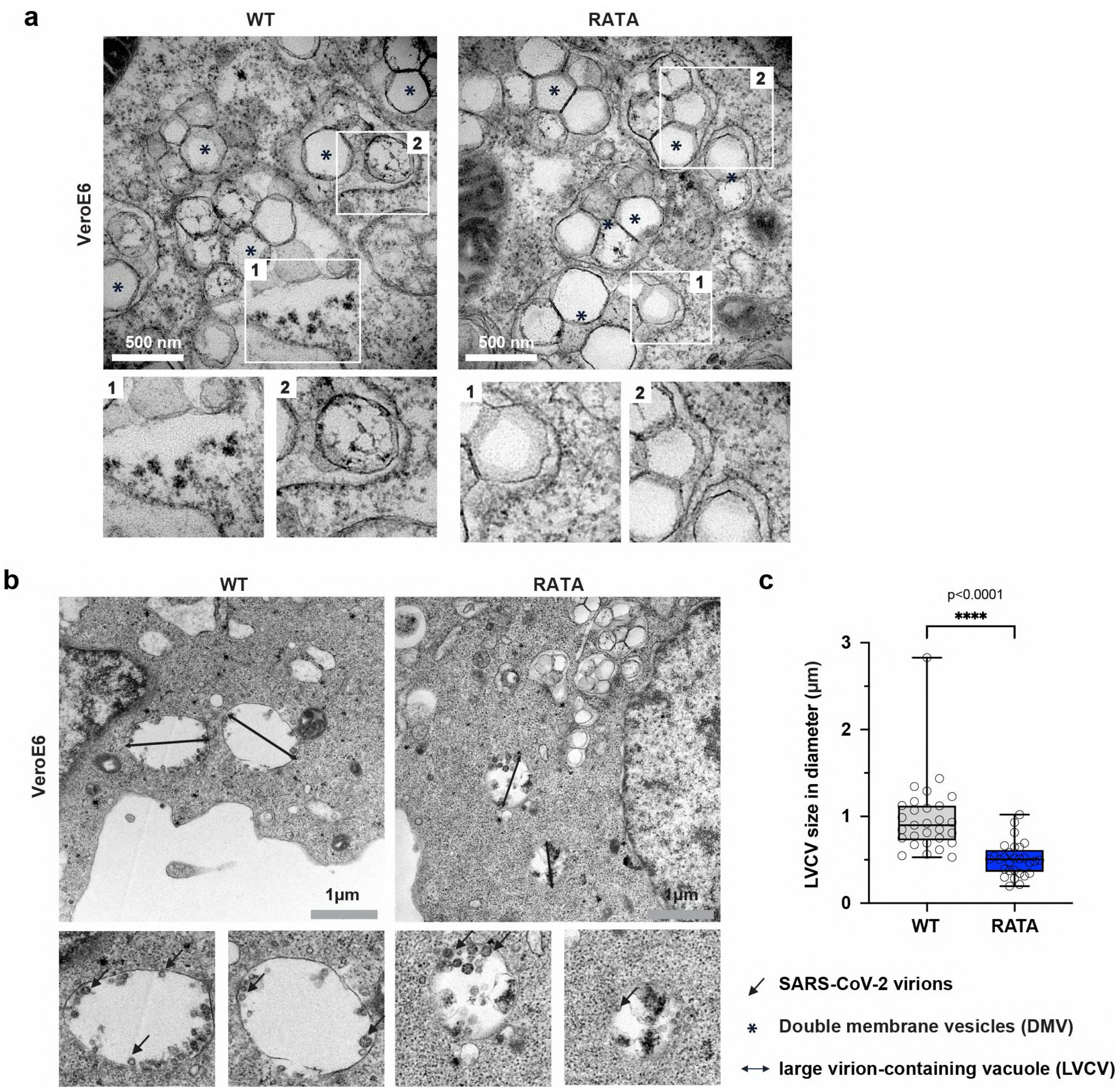

Supplementary Fig. 6. G3BP1 recruits 40S ribosomal subunit to viral factories. (a) VeroE6 cells were infected with SARS-CoV-2 WT or RATA at 0.5 MOI for 10 h and processed for TEM. Scale bar = 500 nm. DMV are indicated with asterisks, (b) and the large virion-containing vacuole (LVCV) with double-headed arrows, black arrow indicates SARS-CoV-2 virions. Scale bar = 1 μm. (c) Analysis of size of LVCV (n=29) in SARS-CoV-2 WT or RATA mutant infected VeroE6 cells.

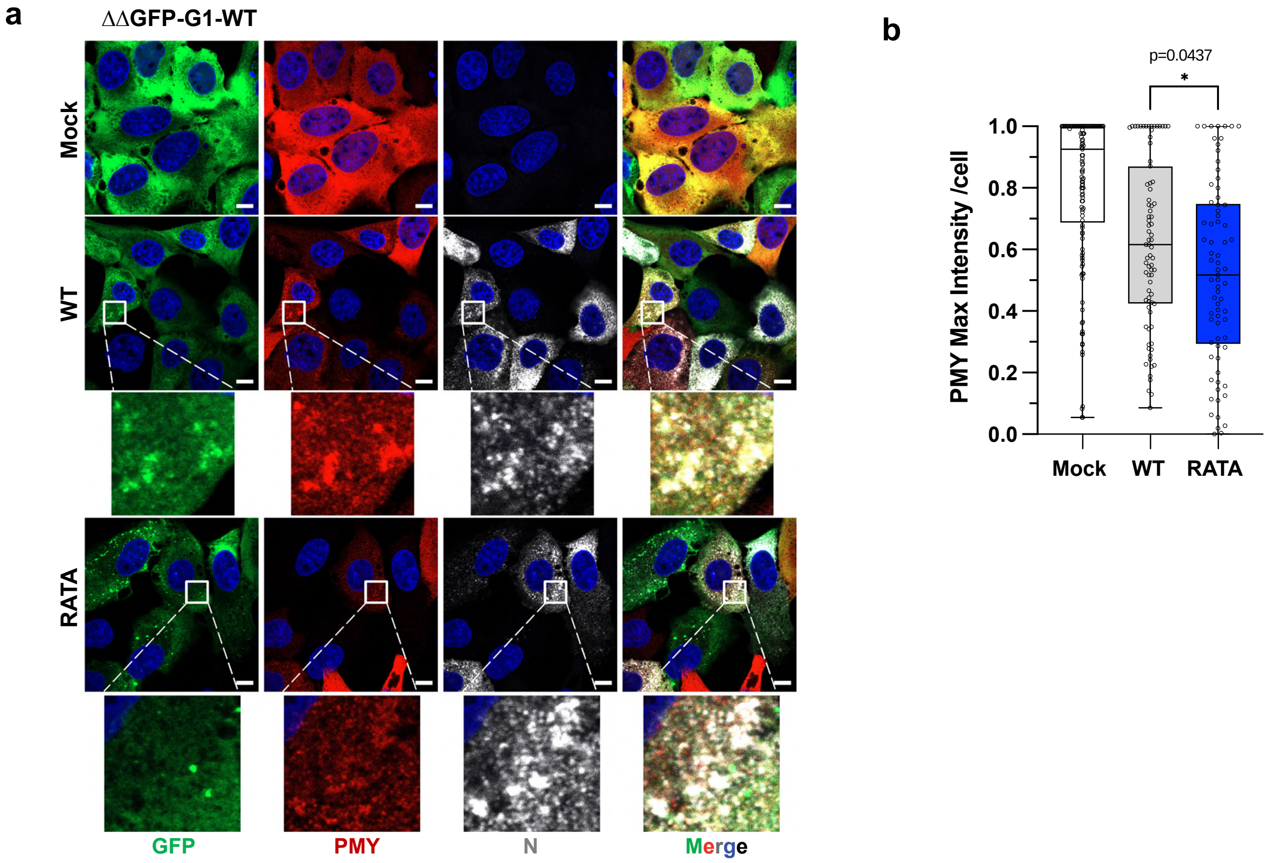

Supplementary Fig. 7. G3BP1 recruits 40S ribosomal subunit to viral factories. (a) ΔΔGFP-G1-WT cells were infected with SARS-CoV-2 WT or RATA at 0.5 MOI for 6 h. Cells were incubated with PMY (20 µg/mL) for 2 min before fixation and stained for PMY (red), N (grey), Hoechst (blue). Representative images from three independent experiments are shown. Scale bar = 10 µm. (b) PMY max intensity were calculated in CellProfiler for n = 125 in mock cells, n=79 in WT-infected cells, n=73 in RATA-infected cells.

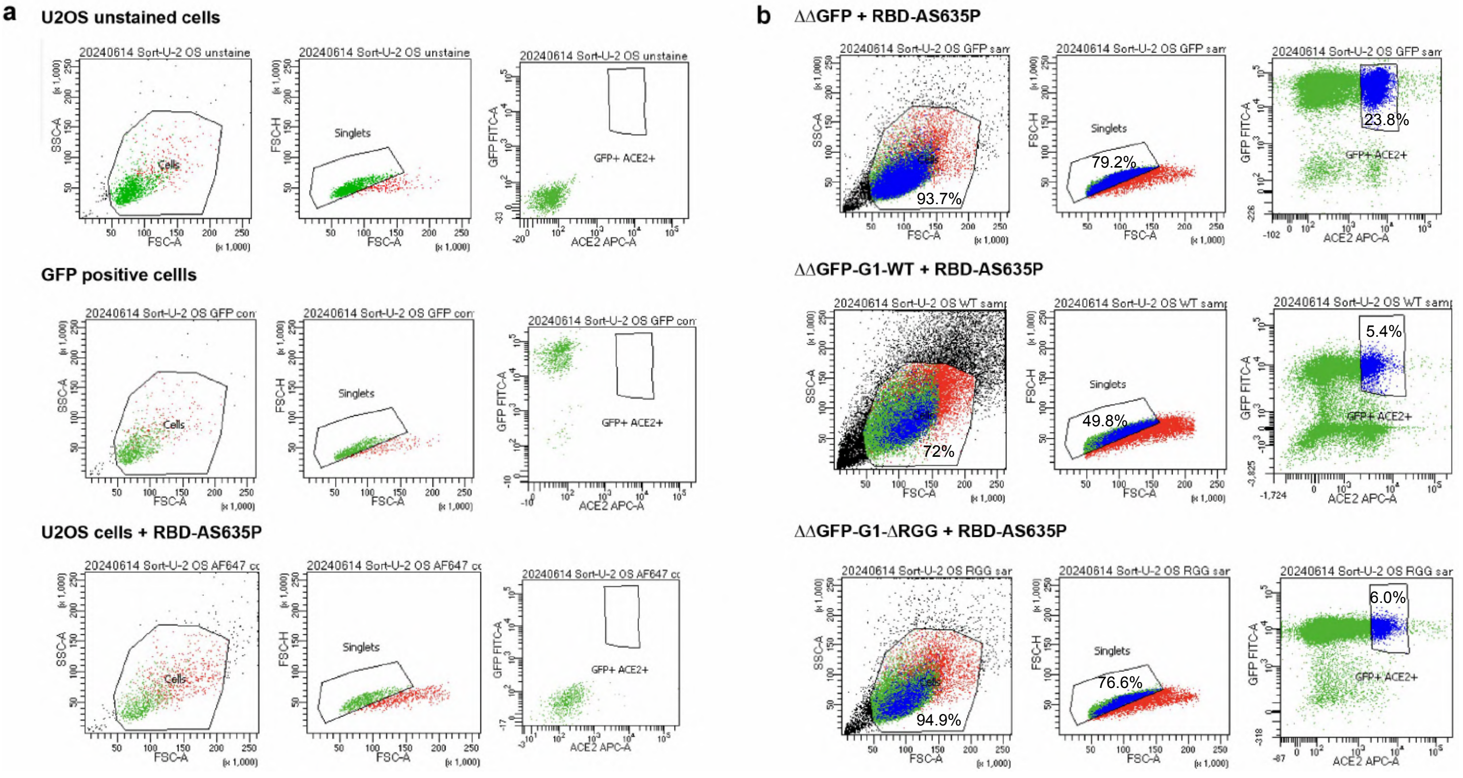

Supplementary Fig. 8. Gating strategy for FACS based on the expression of enhanced green fluorescent protein (GFP) and RBD-AS635P nanobody binding specific to ACE2. (a) "GFP+, ACE+" gating was established using unstained U2OS cells, GFP-positive cells (ΔΔGFP), and U2OS cells stained with RBD-AS635P. (b) The same gate was used to sort "GFP+, ACE+" populations for ΔΔGFP cells, ΔΔGFP-G1-WT cells, or ΔΔGFP-G1-ΔRGG cells, respectively. The percentage of cells within the entire population is indicated in the figure.

Supplementary Table. 1. Primers sequence.

Primer	Sequence	Usage
RATA-F	AAATGCACCCCGCGCTACGGCTGGTGGACCCTCAGATT	Generating pCC1-4K-SARS-CoV-2-RATA plasmid
RATA-R	CGCTGATTTTGGGGTCCATTATCAGACATTTTAGTTTG	
N-seq 1	CAA CCC ATA TGA TGC CGT CTT TG	Sequencing primers as shown in Supplementary 2
N-seq-2	CGAGGACAAGGCGTTCCAATTAAC	
N-seq 3	AAAGATCACATTGGCACCCGC	
N-seq 4	GGGACCAGGAACTAATCAGAC	
IFN-α1-F	AGAAGGCTCCAGCCATCTCTGT	RT-qPCR
IFN-α1-R	TGCTGGTAGAGTTCGGTGCAGA	
IFN-β-F	CTTGGATTCCTACAAAGAAGCAGC	
IFN-β-R	TCCTCCTTCTGGAACTGCTGCA	
GAPDH-F	GTCTCCTCTGACTTCAACAGCG	
GAPDH-R	ACCACCCTGTTGCTGTAGCCAA	
S-F	GAA CAA GAC AAA AAC ACC CAA G	
S-R	GCA ATA TCA CCA AGG CAA TCA C	
N-F	AAGCTGGACTTCCCTATGGTG	
N-R	CGATTGCAGCATTGTTAGCAGG	
E-F	ACAGGTACGTTAATAGTTAATAGCGT	
E-R	ATATTGCAGCAGTACGCACACA	

Supplementary Table. 2. Antibodies list.

Antibody	Company	Cat. No	Dilution
**Immunofluorescence assay**			
G3BP1	ProteinTech	13057-2-AP	1:300
eIF4G	Santa Cruz	sc-133155	1:100
eIF4A	Abcam	ab31217	1:100
SARS-CoV-2 N	OriGene	TA190323	1:500
SARS-CoV-2 nsp3	GeneTex	GTX135589	1:500
dsRNA	SCICONS	10010200	1:200
PMY	Millipore	MABE343	1:500
Alexa Fluor 488	Thermo Fisher Scientific	A21206	1:1000
Alexa Fluor 568	Thermo Fisher Scientific	A10037	1:1000
Alexa Fluor 568	Thermo Fisher Scientific	A10042	1:500
Alexa Fluor 647	Thermo Fisher Scientific	A21445	1:1000
Alexa Fluor 405	Thermo Fisher Scientific	A31553	1:500
Hoechst 33258	Thermo Fisher Scientific	H21491	1:20000
**LLPS**			
Caprin1	ProteinTech	15112-1-AP	1:50
Actin	Santa Cruz	sc-69879	1:500
SARS-CoV-2 N	OriGene	TA190323	1:500
**CO-IP**			
G3BP1	ProteinTech Group	13057-2-AP	1:300
SARS-CoV-2 N	Abcam	ab271180	1:500
GFP	Thermo Fisher Scientific	A6455	1:400
**Western blot**			
PERK	Bioss	BS-2469R-TR	1:1000
p-PERK	Thermo Fisher Scientific	MA5-15033	1:1000
PKR	Abcam	ab184257	1:2000
p-PKR (T446)	Abcam	ab32036	1:1000
eIF2a	Santa Cruz	sc133132	1:1000
eIF2a-s52	Thermo Fisher Scientific	44-728G	1:1000
G3BP1	ProteinTech Group	13057-2-AP	1:2000
G3BP2	ProteinTech Group	16276-1-AP	1:2000
ORF9b	Thermo Fisher Scientific	PA5-116951	1:2000
GAPDH	Santa Cruz	sc-47724	1:1000
SARS-CoV-2 N	Abcam	ab271180	1:2000
SARS-CoV-2 nsp3	GeneTex	GTX135589	1:2000
SARS-CoV-2 Spike	homemade	Verified by PMID:35013189	1:500
