# Supplementary material for: SARS-CoV-2 N protein recruits G3BP to double membrane vesicles to promote translation of viral mRNAs
Source: Nat Commun. 2024 Dec 5;15:10607. doi: 10.1038/s41467-024-54996-3 (PMC11621422; doi:10.1038/s41467-024-54996-3)
Supplement: Supplementary file 3 — Reporting summary [file 41467_2024_54996_MOESM3_ESM.pdf]

nature portfolio

Corresponding author(s): Gerald McInerney

Last updated by author(s): Apr 6, 2024

Reporting Summary

Nature Portfolio wishes to improve the reproducibility of the work that we publish. This form provides structure for consistency and transparency in reporting. For further information on Nature Portfolio policies, see our Editorial Policies and the Editorial Policy Checklist.

Statistics

For all statistical analyses, confirm that the following items are present in the figure legend, table legend, main text, or Methods section.

n/a	Confirmed	
☐	☒	The exact sample size (*n*) for each experimental group/condition, given as a discrete number and unit of measurement
☐	☒	A statement on whether measurements were taken from distinct samples or whether the same sample was measured repeatedly
☐	☒	The statistical test(s) used AND whether they are one- or two-sided<br>*Only common tests should be described solely by name; describe more complex techniques in the Methods section.*
☒	☐	A description of all covariates tested
☒	☐	A description of any assumptions or corrections, such as tests of normality and adjustment for multiple comparisons
☐	☒	A full description of the statistical parameters including central tendency (e.g. means) or other basic estimates (e.g. regression coefficient) AND variation (e.g. standard deviation) or associated estimates of uncertainty (e.g. confidence intervals)
☐	☒	For null hypothesis testing, the test statistic (e.g. *F*, *t*, *r*) with confidence intervals, effect sizes, degrees of freedom and *P* value noted<br>*Give P values as exact values whenever suitable.*
☒	☐	For Bayesian analysis, information on the choice of priors and Markov chain Monte Carlo settings
☒	☐	For hierarchical and complex designs, identification of the appropriate level for tests and full reporting of outcomes
☐	☒	Estimates of effect sizes (e.g. Cohen's *d*, Pearson's *r*), indicating how they were calculated

Our web collection on statistics for biologists contains articles on many of the points above.

Software and code

Policy information about availability of computer code

Data collection	No software was used for the collection of data in this study
Data analysis	Image Lab 6.0.1 (https://www.bio-rad.com/en-se/product/image-lab-software?ID=KRE6P5E8Z)<br>GraphPad Prism 10 (https://www.graphpad.com/updates/prism-1000-release-notes)<br>Image J 1.52q (http://imagej.nih.gov/ij)<br>Fiji (https://imagej.net/software/fiji/downloads)<br>CellProfiler 4.2.6 (https://cellprofiler.org/releases)<br>Zen 2 (Blue) v2.3 (https://www.zeiss.com/microscopy/en/products/software/zeiss-zen.html)<br>PyMol 2.5.4 (https://pymol.org/)<br>FlowJo v10(https://www.flowjo.com/)

For manuscripts utilizing custom algorithms or software that are central to the research but not yet described in published literature, software must be made available to editors and reviewers. We strongly encourage code deposition in a community repository (e.g. GitHub). See the Nature Portfolio guidelines for submitting code & software for further information.

Data

Policy information about availability of data

All manuscripts must include a data availability statement. This statement should provide the following information, where applicable:

- Accession codes, unique identifiers, or web links for publicly available datasets
- A description of any restrictions on data availability
- For clinical datasets or third party data, please ensure that the statement adheres to our policy

The study involves no large datasets that should be uploaded to any depository. Raw data from our experiments can be found in the Source Data files.

Research involving human participants, their data, or biological material

Policy information about studies with human participants or human data. See also policy information about sex, gender (identity/presentation), and sexual orientation and race, ethnicity and racism.

Reporting on sex and gender	N/A
Reporting on race, ethnicity, or other socially relevant groupings	N/A
Population characteristics	N/A
Recruitment	N/A
Ethics oversight	N/A

Note that full information on the approval of the study protocol must also be provided in the manuscript.

Field-specific reporting

Please select the one below that is the best fit for your research. If you are not sure, read the appropriate sections before making your selection.

☒ Life sciences ☐ Behavioural & social sciences ☐ Ecological, evolutionary & environmental sciences

For a reference copy of the document with all sections, see nature.com/documents/nr-reporting-summary-flat.pdf

Life sciences study design

All studies must disclose on these points even when the disclosure is negative.

Sample size	Sample size for all experiments are detailed in relevant text or/and figure legend.
Data exclusions	No
Replication	For all experiments, a minimal of three independent experiments were performed as indicated in the figure legends. All attempts at replication were successful.
Randomization	All animals were randomly assigned to experimental groups to this study.
Blinding	In most studies, investigators were not blinded because the readouts were objective.

Reporting for specific materials, systems and methods

We require information from authors about some types of materials, experimental systems and methods used in many studies. Here, indicate whether each material, system or method listed is relevant to your study. If you are not sure if a list item applies to your research, read the appropriate section before selecting a response.

Materials & experimental systems

n/a	Involved in the study
☐	☒ Antibodies
☐	☒ Eukaryotic cell lines
☒	☐ Palaeontology and archaeology
☐	☒ Animals and other organisms
☒	☐ Clinical data
☒	☐ Dual use research of concern
☒	☐ Plants

Methods

n/a	Involved in the study
☒	☐ ChIP-seq
☐	☒ Flow cytometry
☒	☐ MRI-based neuroimaging

Antibodies

Antibodies used	anti-G3BP1-rabbit-polyclonal (ProteinTech Group, Cat No :13057-2-AP, Lot: 00108267);<br>anti-eIF4G-mouse-monoclonal (Santa Cruz, Cat No: sc-133155, Lot: K0422);<br>anti-eIF4A-rabbit-polyclonal (Abcam, ab31217);<br>anti-SARS-CoV-2 N-human- monoclonal (OriGene, Cat No: TA190323, Lot: RH001, Clone: OT1H1G5);<br>anti-nsp3-rabbit-polyclonal (GeneTex, Cat No: GTX135589, Lot: 44062);<br>anti-dsRNA-mouse-monoclonal (SCICONS, Cat No: 10010200, Lot: 18542, Clone: J2);<br>anti-PMY-mouse-monoclonal (Millipore, Cat No: MABE343, Lot: 2861354, Clone: 12D10);<br>anti-SARS-CoV-2 N-rabbit-monoclonal (Abcam, Cat No: ab271180, Lot: 1015927-4);<br>anti-GFP-rabbit-polyclonal (Thermo Fisher Scientific, Cat No: A6455, Lot: 1964399);<br>anti-PERK-rabbit-polyclonal (Bioss, Cat No: BS-2469R-TR, Lot: AI08071172);<br>anti-p-PERK-rabbit-monoclonal (Thermo Fisher Scientific, Cat No: MA5-15033, Lot: YG3988242);<br>anti-PKR-rabbit-monoclonal (Abcam, Cat No: ab184257, Lot:1006611-7);<br>anti-p-PKR-rabbit-monoclonal (T446) Abcam Cat No: ab32036, Lot: 1007369-9, Clone: E120);<br>anti-eIF2a-mouse-monoclonal (Santa Cruz, Cat No: sc133132, Lot: J1419, Clone: D-3);<br>anti-eIF2a-s52-rabbit-polyclonal (Thermo Fisher Scientific, Cat No: 44-728G, Lot: 2548959);<br>anti-G3BP2-rabbit-polyclonal (ProteinTech Group, Cat No: 16276-1-AP, Lot: 00079746);<br>anti-ORF9b-rabbit-polyclonal (Thermo Fisher Scientific Cat No: PA5-116951 Lot: XF3627697A);<br>anti-GAPDH-mouse-monoclonal (Santa Cruz, Cat No: sc-47724, Lot: A0721, Clone, 0411);<br>Alexa Fluor 488 donkey anti-mouse IgG (H+L) (Thermo Fisher Scientific, Cat No: A21206, Lot: 2147618);<br>Alexa Fluor 568 donkey anti-mouse IgG (H+L) (Thermo Fisher Scientific, Cat No: A10037, Lot: 2420698);<br>Alexa Fluor 568 donkey anti-rabbit IgG (H+L) (Thermo Fisher Scientific, Cat No: A10042 Lot: 2306809);<br>Alexa Fluor 647 goat anti-human IgG (H+L) (Thermo Fisher Scientific, Cat No: A21445, Lot: 2491370);<br>Alexa Fluor 405 goat anti-mouse IgG (H+L) (Thermo Fisher Scientific, Cat No: A31553, Lot: 2306799);<br>Hoechst 33258 (Thermo Fisher Scientific, Cat No: H21491, Lot: 1050083);<br>Anti-rabbit IgG, HRP-linked (Cell signaling technology, Cat No: 7074S, Lot: 32);<br>Anti-mouse IgG, HRP-linked (Thermo Fisher Scientific, Cat No: A9044, Lot: 055M4818V);<br>Homemade Spike.
Validation	We only use antibodies that are commercially available and have been validated by different companies. For more information about antibody validation by different brands visit https://www.sigmaaldrich.com/SE/en; https://www.abcam.com/en-se; https://www.thermofisher.com/se/en/home.html; https://www.cellsignal.com/; https://www.scbt.com/home; https://www.ptglab.com/; https://www.origene.com/; https://www.genetex.cn/; https://www.biossusa.com/; Homemade Spike nanobody was v.erified by PMID:35013189.

Eukaryotic cell lines

Policy information about cell lines and Sex and Gender in Research

Cell line source(s)	Cell lines were purchased from ATCC: HEK293T (ATCC-CRL-3216, female), U2OS cells 331 (ATCC HTB-96, female), VeroE6 (ATCC CRL-1586), MA104 (ATCC-CRL-2378), BHK-21 cells (ATCC CCL-10).
Authentication	Authentication was not performed, since all lines were recently purchased from ATCC.
Mycoplasma contamination	All cell lines used for experiments were negative for mycoplasma as determined by specific PCR assay.
Commonly misidentified lines<br>(See ICLAC register)	No commonly misidentified cell lines were used in the study.

Animals and other research organisms

Policy information about studies involving animals; ARRIVE guidelines recommended for reporting animal research, and Sex and Gender in Research

Laboratory animals	K18-hACE2 transgenic mice (Jackson Laboratories) were were 18-20 weeks old at the start of the study.

Wild animals	This study did not involve wild animals
Reporting on sex	All mice were male. Male K18-hACE2 mice generally have slighter higher body weight than females, but SARS-CoV-2 infection-induced weight loss is similar.
Field-collected samples	This study did not involve samples collected in the field.
Ethics oversight	Ethical permits for studies of virus infection were obtained from the Swedish Board of Agriculture (10513-2020)

Note that full information on the approval of the study protocol must also be provided in the manuscript.

Plants

Seed stocks	N/A
Novel plant genotypes	N/A
Authentication	N/A

Flow Cytometry

Plots

Confirm that:

☒ The axis labels state the marker and fluorochrome used (e.g. CD4-FITC).

☒ The axis scales are clearly visible. Include numbers along axes only for bottom left plot of group (a 'group' is an analysis of identical markers).

☐ All plots are contour plots with outliers or pseudocolor plots.

☒ A numerical value for number of cells or percentage (with statistics) is provided.

Methodology

Sample preparation	Cells were trypsinized and stained with or without RBD-AS635P.
Instrument	BD FACSDiva 9.0.1
Software	FlowJo
Cell population abundance	The percent of single cells and the percent of "GFP+, ACE+" within the whole population were indicated in Supplementary. Fig.8b.
Gating strategy	FSC-A and SSC-A were used to identify all cells, and FSC-A and FSC-H were used to identify single cells. Gating was performed using unstained U2OS cells, GFP-positive cells (U2OSΔΔGFP), and U2OS cells stained with RBD-AS635P to identify 'GFP-positive and ACE2-positive cells

☒ Tick this box to confirm that a figure exemplifying the gating strategy is provided in the Supplementary Information.
